# Comparative effectiveness of light emitting diodes (LEDs) and Lasers in near infrared photoimmunotherapy

**DOI:** 10.18632/oncotarget.7365

**Published:** 2016-02-13

**Authors:** Kazuhide Sato, Rira Watanabe, Hirofumi Hanaoka, Takahito Nakajima, Peter L. Choyke, Hisataka Kobayashi

**Affiliations:** ^1^ Molecular Imaging Program, Center for Cancer Research, National Cancer Institute, Bethesda, MD, USA

**Keywords:** near infrared photoimmunotherapy, light emitting diode (LED), light amplification by stimulated emission of radiation (Laser), epidermal growth factor receptor, super-enhanced permeability and retention (SUPR) effect

## Abstract

Near infrared photoimmunotherapy (NIR-PIT) is a new cancer treatment that combines the specificity of antibodies for targeting tumors with the toxicity induced by photosensitizers after exposure to near infrared (NIR) light. Herein we compare two NIR-light sources; light emitting diodes (LEDs) and Lasers, for their effectiveness in NIR-PIT.

A photosensitizer, IRDye-700DX, conjugated to panitumumab (pan-IR700), was incubated with EGFR-expressing A431 and MDA-MB-468-luc cells. NIR-light was provided by LEDs or Lasers at the same light dose. Laser-light produced more cytotoxicity and greater reductions in IR700-fluorescence intensity than LED-light. Laser-light also produced more cytotoxicity *in vivo* in both cell lines. Assessment of super-enhanced permeability and retention (SUPR) effects were stronger with Laser than LED.

These results suggest that Laser-light produced significantly more cytotoxic effects compared to LEDs. Although LED is less expensive, Laser-light produces superior results in NIR-PIT.

## INTRODUCTION

Both light emitting diodes (LEDs) and Lasers are now widely used in many clinical fields [1, 2]. Although LEDs can irradiate wider fields and are less expensive than Lasers, they have broader bandwidth and more variable wavelenths, amplitude and phase, compared to Laser-ight. On the other hand, although Laser-light has high coherency, monochromatic wavelength, and stability, it costs substantially more and has a narower beam than LEDs [3, 4]. Therefore, the choice of light source for specific medical applications is a challenge.

Near infrared photoimmunotherapy (NIR-PIT) is a new cancer treatment based on an antibody-photosensitizer conjugate (APC) [5]. NIR-PIT conjugates combine the specificity of antibodies with the toxicity induced by photosensitizers after exposure to NIR-light. IRDye-700DX (IR700, a silica-phthalocyanine dye) is a highly hydrophilic photosensitizer that is excited by NIR-light at a wavelength of 690 nm. *In vitro* studies have shown NIR-PIT is highly cell-specific, with high levels of cytotoxicity in antigen expressing cells and virtually no cytotoxic effects in immediately adjacent non-expressing cells [6–8]. Recent data suggests that once the APC binds to the target cell and is exposed to NIR-light, it quickly causes irreversible damage to the cell membrane [9]. Within minutes of exposure to NIR-light, the membrane ruptures leading to cell death in a highly selective manner [10, 11]. While this is a promising treatment, it is still unclear which method of delivering light, LED or Laser, is superior. As NIR-PIT enters clinical trials, this question becomes more important.

In this study, we compare the *in vitro* and *in vivo* cytotoxic efficacy of NIR-PIT using either LED or Laser-NIR-light.

## RESULTS

### Overview of LED/Laser, and evaluation of decrease of IR700-fluorescence

**Figure 1:**
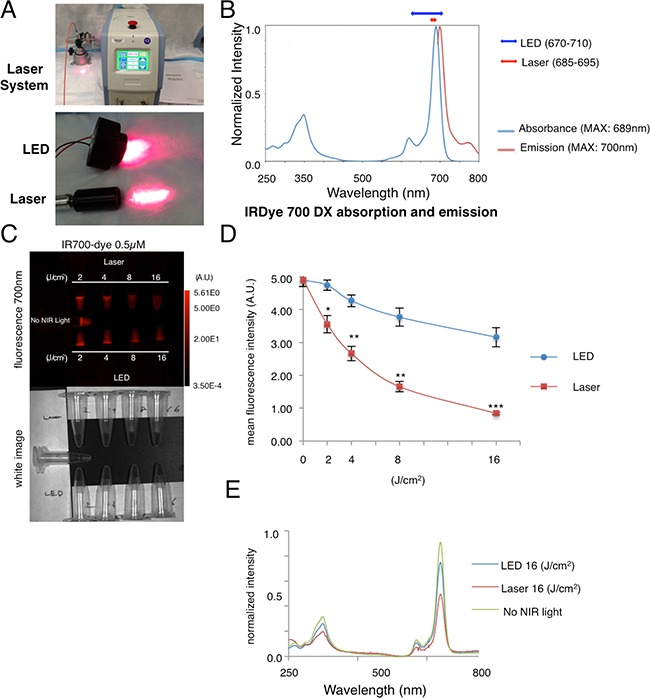
Overview of LED/Laser devices, and decrease in IR700-fluorescence induced by either LED or Laser (**A**) The NIR Laser system, and LED system used in this study. (**B**) Character of wavelength of LED and Laser-light in reference to the absorption of IR700 dye (**C**) The decrease in IR700-fluorescence was greater with Laser than LED at the same energy level (upper: fluorescence 700 nm, lower: white image). The decrease of IR700-fluorescence was detected in a dose dependent manner. (**D**) Quantification of fluorescence mean intensity showed a larger decrease with Laser-light than LED at the same dose (*n* = 5, *P < 0.01, **P < 0.001, ***P < 0.0001). (**E**) A greater decrease of IR700-fluorescence after Laser-light vs. LED-light was detected with spectroscopy.

The characteristics of NIR-light produced by LED and Laser are shown (Figure 1A). The bandwidth of Laser-light is narrower than that of LED (Figure 1B). Using the same light dose (measured in J/cm^2^) the effects of NIR-light after LED and Laser-light were compared. IR700-fluorescence was evaluated in IR700 solutions, and quantified (Figure 1C, 1D). The IR700-fluorescence intensity decreased in a dose dependent manner (Figure 1C), which was quantified by mean fluorescence intensity (Figure 1D). Laser-light resulted in more decrease of IR700-fluorescence than LED-light (Figure 1E) due to photo-bleaching or photo-chemical reaction. These data suggest that Laser-light induced more decrease of IR700-fluorescence than did LED at the same dose (J/cm^2^).

### Laser-light produces more NIR-PIT-induced cytotoxicity than LED-light in 2D and 3D spheroid cultures

**Figure 2:**
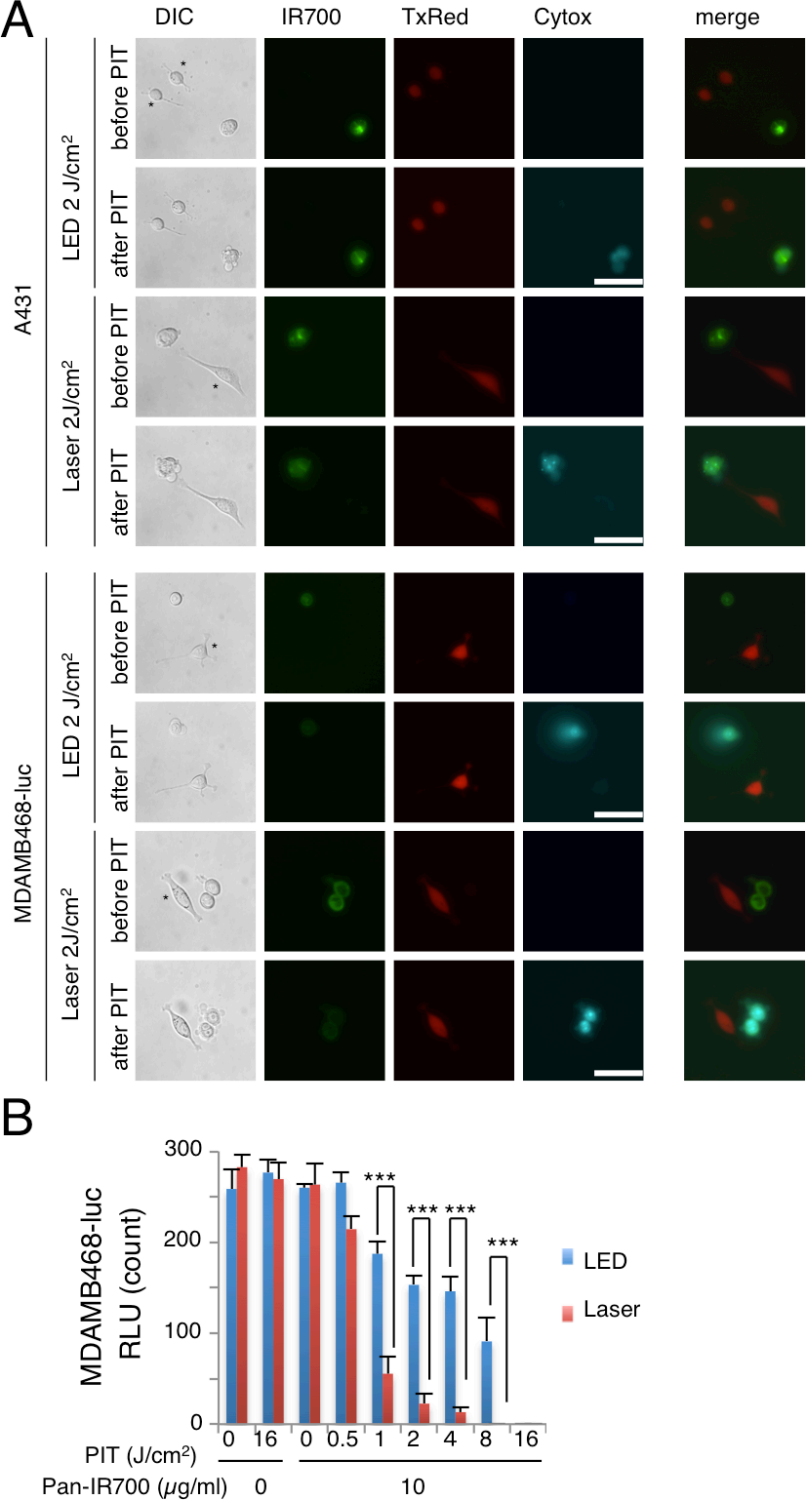
*In vitro* NIR-PIT effect with LED or Laser (**A**) A431 and MDA-MB-468-luc cells were co-cultured with 3T3/DsRed (non-HER expressing) cells. They were treated with pan-IR700 and observed by microscopy (before and after irradiation with either Laser or LED NIR-light. Target specific necrotic cell death was observed after excitation with NIR-light (after 30 min). No damage was demonstrated in 3T3/DsRed cells with LED or Laser. *3T3/DsRed cells, Bar = 50 μm. (**B**) Luciferase activity in MDA-MB-468-luc cells decreased after both LED and Laser mediated NIR-PIT in a light dose-dependent manner. Significant differences between LED and Laser were detected. (*n* = 4; ***P < 0.01).

Serial fluorescence microscopy was performed after NIR-PIT using LED or Laser to examine their comparative *in vitro* effects. Immediately after exposure to NIR-light (2 J/cm^2^) cellular swelling, bleb formation was observed in both A431 and MDA-MB-468-luc cells (Figure 2A). Most of these cellular changes were observed within 30 min of light exposure, indicating rapid induction of necrotic cell death after NIR-PIT with both light sources. No significant differences in non EGFR-expressing 3T3 cells (3T3/DsRed) after irradiation with either light source was observed.

In order to examine the effects of *in vitro* NIR-PIT quantitatively, we performed a cytotoxicity assay based on luciferase activity. The luciferase activity assay in MDA-MB-468-luc cells showed significant decreases of relative light units (RLU) related to NIR-PIT-induced reductions in ATP production in living cells, indicating a decrease in cellular activity which was light dose dependent (Figure 2B). Significant differences were also detected between treatments with LED and Laser. These studies suggest that Laser-light produces more cytotoxicity at the same energy level than LED-light.

**Figure 3:**
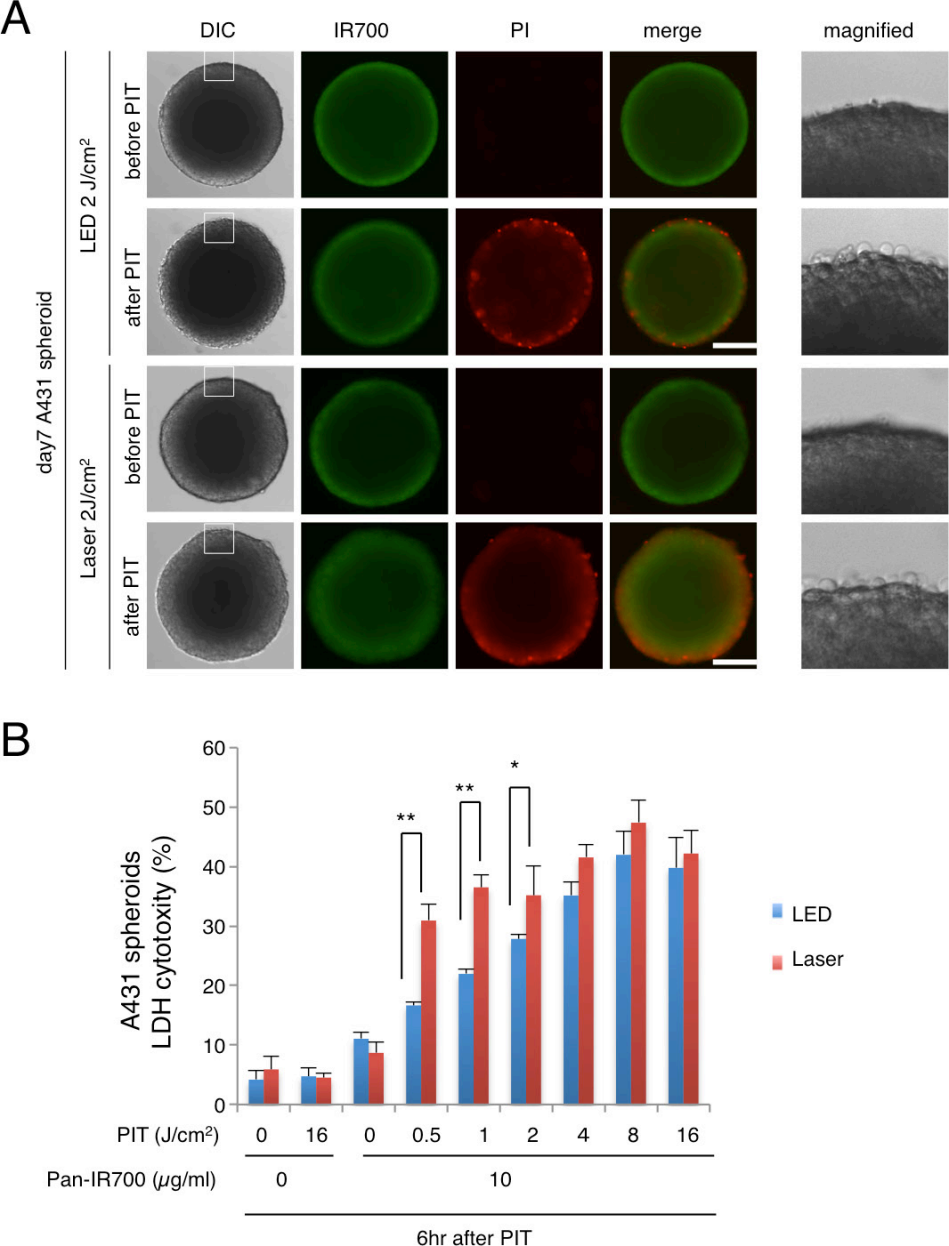
LED vs Laser NIR-PIT in *in vitro* 3D spheroids (**A**) A431 3D spheroids treated with NIR-PIT mediated by LED or Laser were observed by microscopy (before and after irradiation of NIR-light). Necrotic cell damage was observed after NIR-PIT. Bar = 100 μm. (**B**) LDH cytotoxicity assay (for the spheroids treated with NIR-PIT mediated by LED or Laser) showed increasing cell death with increases in light dose. Significant differences in efficacy were demonstrated between LED and Laser (*n* = 5; *P < 0.05, **P < 0.01).

The efficacy of *in vitro* NIR-PIT was also examined with A431 3D spheroids. To visualize and quantify the effects of NIR-PIT in the 3D spheroid model, concurrent microscopic observation and the Lactate dehydrogenase (LDH) cytotoxicity assay were performed. At 1 hr post-NIR-PIT, there was physical swelling of the spheroids (Figure 3A). The outer layer of the spheroid was stained with Propidium Iodide, indicating cell death where NIR-light penetrated, and the thickness of the dead cell layer was deeper with Laser than LED. The LDH cytotoxicity assay showed significant cell death that was light dose dependent but an absence of cell death without agents or light exposure and, a significant difference between treatments with LED and Laser were detected (Figure 3B). These results revealed that Laser-light resulted in greater cytotoxicity than LED-light after NIR-PIT both in 2D and 3D cell cultures.

### Laser has superior efficacy to LED for *in vivo* NIR-PIT

**Figure 4:**
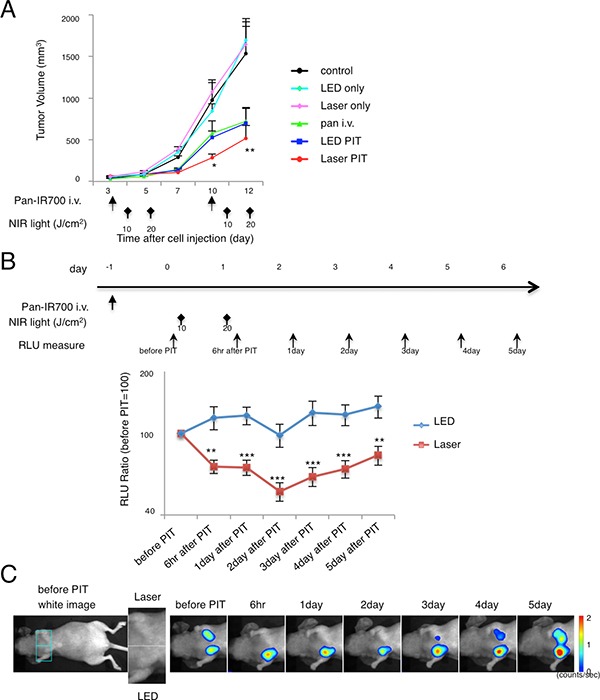
NIR-PIT with Laser showed superior *in vivo* anti-tumor effects (**A**) Repeated Laser NIR-PIT led to more effective A431 tumor volume reduction than LED (Laser PIT group vs. LED PIT group at day 10: *p = 0.0175 < 0.05) (*n* = 10 mice in each treatment group; Laser PIT group or LED PIT group vs. control at day 12: **P = 0.0002 < 0.001, by Kruskal-Wallis test with post-test). The treatment regimen is shown below the graph. (**B**) Repeated NIR-PIT with Laser lead to lower bioluminescence activity within the tumor compared with LED (*n* = 10 mice in each treatment group; **P < 0.01, ***P < 0.05). The treatment regimen and time point of measurement of bioluminescence are indicated above the graph. (**C**) Bioluminescence images (BLI) of MDA-MB-468-luc orthotopic tumors in response to repeated NIR-PIT with LED or Laser. Typical images of a mouse that had almost the same RLU in both tumor regions before therapy is depicted. Laser irradiation was performed on the left side orthotopic tumor while LED irradiation was performed on the right side.

We compared the two light sources for their *in vivo* NIR-PIT effects using A431 xenografts and orthotopically grafted MDA-MB-468-luc tumors at the same light dose (J/cm^2^). Significant differences in the efficacy of NIR-PIT after Laser or LED were observed in A431 xenograft mice (Figure 4A). Tumor volume was reduced significantly in the Laser NIR-PIT group compared with the LED NIR-PIT group (*n* = 10 mice in each group)(tumor volume; LED PIT group vs Laser PIT group at day10: *p* = 0.0175 < 0.05 (Kruskal-Wallis test with post-test)). Mice irradiated with either LED or Laser alone showed no tumor growth inhibition. Mice injected with pan-IR700 alone showed minimal tumor growth inhibition. We also examined the therapeutic effect of *in vivo* NIR-PIT by bioluminescence quantification (Figure 4B, 4C). The RLU ratio, (post-PIT RLU to pre-PIT RLU), in MDA-MB-468-luc orthotopic breast tumors demonstrated that Laser exposed animals had significant decreases in RLU ratio at 6 hr and 1 day after NIR-PIT (**p < 0.05, ***p < 0.01) (Figure 4C). The RLU ratio in the LED NIR-PIT group temporally decreased at 2 day after NIR-PIT probably due to the accumulated damage to the tumors. On the other hand, in the Laser group, the RLU decreased until 2 day after NIR-PIT. BLI showed visible differences in luciferase activity within the same mouse (Figure 4C). Significant differences between LED and Laser groups were detected at all time points (Figure 4B). Thus, Laser resulted in more effective *in vivo* NIR-PIT than did LED.

**Figure 5:**
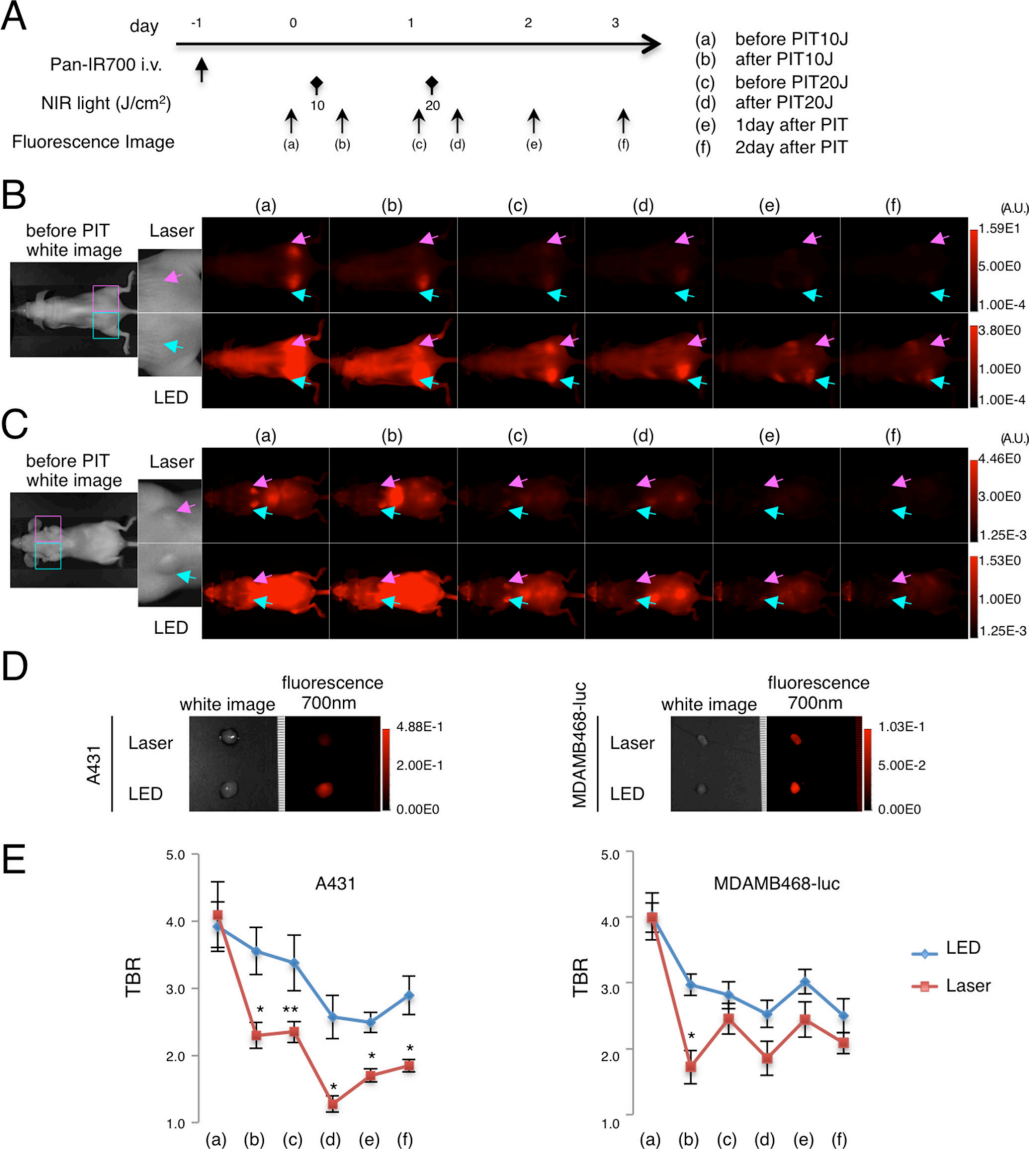
*In vivo* fluorescence imaging in response to repeated LED or Laser mediated NIR-PIT Fluorescence images were obtained at each time point before and after repeated NIR-PIT as indicated (**A**). *In vivo* fluorescence imaging of A431 xenografts (left for LED, right for Laser) (**B**) and MDA-MB-468-luc orthotopic breast tumors (right for LED, left for Laser) (**C**) tumor bearing mice treated with repeated NIR-PIT showed differences in fluorescence signal after LED and Laser irradiation (upper; high threshold, lower; low threshold). Tumors of almost the same size were selected for comparison (see magnified view). *Ex vivo* fluorescence images of A431 or MDA-MB-468-luc tumor (**D**) at 2 days after NIR-PIT also showed lower intensity with Laser than with LED. Tumor-to-background ratio (TBR) of the IR700-fluorescence intensity in A431 tumors or MDA-MB-468-luc orthotopic breast tumors (**E**) demonstrated quantitative differences in fluorescence (*n* = 10 mice in each treatment group; *P < 0.01, **P < 0.05).

To further elucidate the difference in *in vivo* effects of NIR-PIT between LED and Laser, serial fluorescence images of the tumor-bearing mice were assessed (Figure 5A). After NIR-PIT, the tumor irradiated by Laser (right dorsum in A431 tumor bearing mouse/left breast orthotopic tumor in MDA-MB-468-luc mouse) demonstrated lower IR700-fluorescence intensity than the tumor irradiated by LED in both A431 and MDA-MB-468-luc tumors (Figure 5B, 5C). *Ex vivo* analysis confirmed lower IR700-fluorescence intensity in the Laser-irradiated tumor than the LED irradiated tumor (Figure 5D). Tumor-to-background-ratio (TBR) also indicated more decrease of IR700-fluorescence in tumors irradiated by Laser compared to LED (Figure 5E). Intriguingly, 1 day after the first NIR-PIT treatment (*i.e.* before the second PIT treatment), higher recovery of TBR was seen in the Laser group in both tumor models, but especially in the MDA-MB-468-luc mouse model (Figure 5E (c)). These results suggested that Laser-light not only resulted in higher efficacy *in vivo* and *in vitro*, but also induced more leakage of circulating pan-IR700 into tumors after the first NIR-PIT.

### Laser-light resulted in stronger SUPR effects

**Figure 6:**
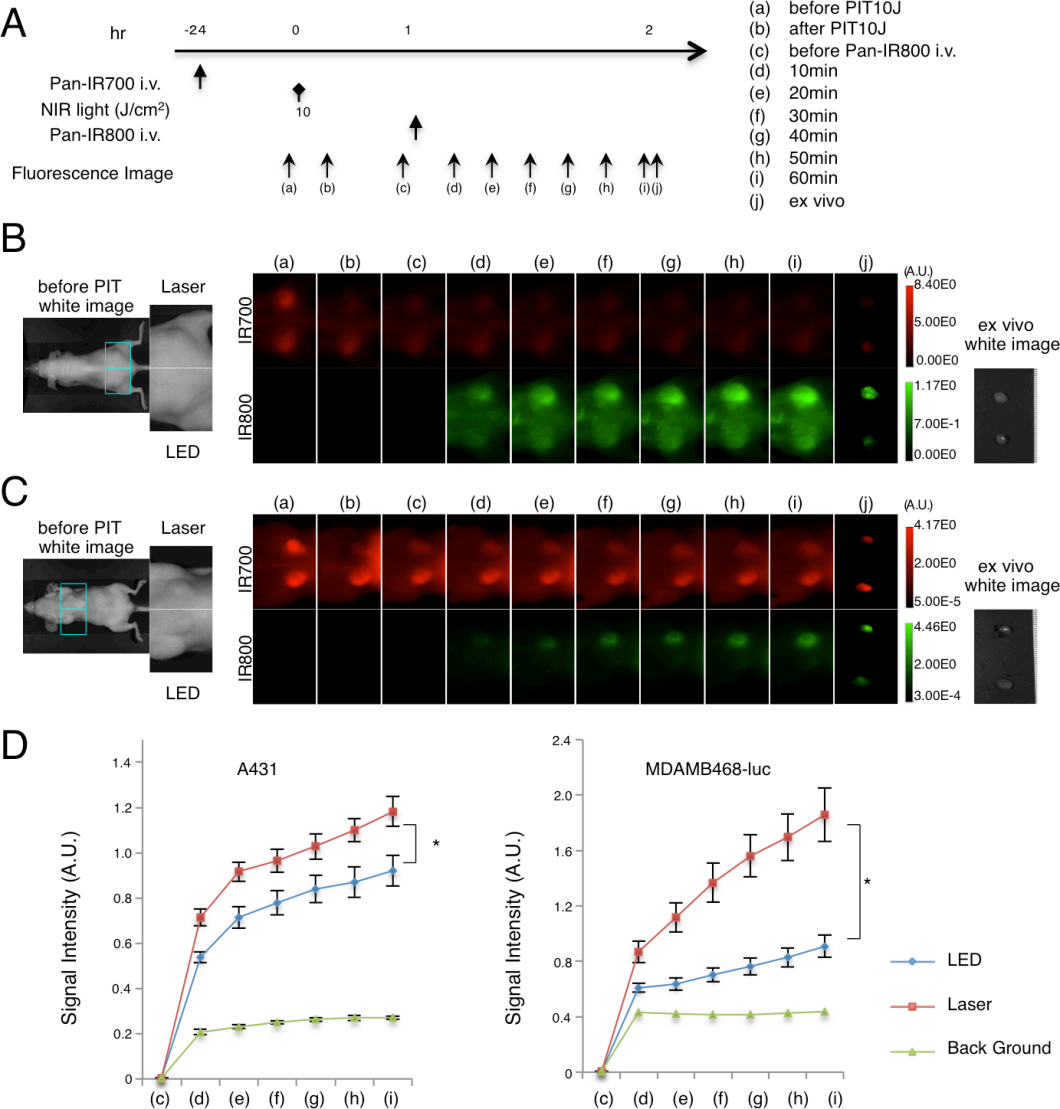
Stronger SUPR effects are induced by Laser mediated NIR-PIT than LED The SUPR effect was evaluated by measuring fluorescence signals of pan-IR800 injected 1 day after NIR-PIT using pan-IR700 at each time point (**A**). *In vivo* fluorescence imaging of A431 xenograft (left for LED, right for Laser) (**B**) and MDA-MB-468-luc orthotopic breast tumor (right for LED, left for Laser) (**C**) tumor bearing mice evaluated for the SUPR effect showed differences in fluorescence signal between treatments with LED and Laser (upper; IR700, lower; IR800, SUPR effect). Tumors of the same size were selected (see magnified view). (**D**) IR800 signal intensity (IR800-SI) of NIR-PIT-treated tumors and background. IR800-SI in A431 tumors or MDA-MB-468-luc orthotopic breast tumors treated with LED or Laser showed stronger signals in Laser treated tumors (*n* = 10 mice in each treatment group; *P < 0.0001).

The higher recovery of fluorescence of pan-IR700 after the first NIR-PIT is indicative of a stronger super-enhanced permeability and retention (SUPR) effect induced by Laser than LED [12]. To examine the differences in the SUPR effect induced by either LED or Laser, serial IR800-fluorescence imaging was performed with injection of pan-IR800 after NIR-PIT in EGFR-positive tumor-bearing mice (Figure 6A). Both A431 and MDA-MB-468-luc tumors showed higher IR800-fluorescence intensity with Laser than LED at all time points (Figure 6B, 6C). With *ex vivo* imaging at 60 min after injection, higher IR800 fluorescence signals (IR800-SI) were observed within tumors following Laser irradiation than following LED irradiation (Figure 6B (j), 6C (j)). Quantification of IR800-SI in both tumors showed higher intensity in the Laser group than the LED group (Figure 6D) (*n* = 10 mice in each group, *P < 0.0001). The effect of Laser on the SUPR effect was greater in MDA-MB-468-luc tumors than A431 tumors, consistent with the higher recovery in TBR of IR700-fluorescence (Figure 5E). These results suggest that NIR-PIT induced by Laser-light has a stronger SUPR effect than LED-light in tumor bearing mice and, that the stronger SUPR effect likely contributed to the higher efficacy of NIR-PIT with Laser *in vivo*.

## DISCUSSION

LEDs and Lasers are both widely used in many clinical fields and are both viable options for NIR-PIT [3]. We show that NIR-PIT with Laser-light had superior cytotoxic efficacy to LED-light at the same energy levels in both 2D and 3D-spheroid cell cultures *in vitro*. Laser-light resulted in superior therapeutic effects compared to LED-light in *in vivo* NIR-PIT at the same light dose in mouse models. Furthermore Laser-light induced stronger SUPR effects in *in vivo* mouse models than LED-light. The spectrum of Laser-light is fit better to IR700 absorbance spectrum than that of LED-light (Figure 1B), therefore, Laser-light is absorbed more effectively to IR700. These data suggest that Laser-light would be preferred over LED-light in NIR-PIT therapeutic applications. Moreover, many oncologic uses of NIR-PIT require delivery of light via catheters, endoscopes, drainage tubes, and needles etc. Therefore, a light source with a narrow beam, such as that produced by coherent Laser-light is preferred. The higher light fluency of Lasers could shorten the exposure time, resulting in more efficient treatments by NIR-PIT. Shorter irradiation time is ideal, because patients have to keep their body still during the therapy. Additionally, LED exposes more unabsorbed light that deposit energy for heating up the tissue than Laser. When applying appropriate light fluency, toxicity of Laser-light would be minimal, because cytotoxic effects of NIR-PIT is highly target cell selective and Laser-light locally irradiates only to treating regions.

An important aspect of NIR-PIT is that after the first exposure to NIR-light, there are rapid and dramatic increases in vascular permeability to nano-sized molecules such as antibodies [12, 13]. NIR-PIT only targets tumor cells and thus the vessels remain intact so that blood flow is maintained. Moreover, the first cells to be killed by NIR-PIT are perivascular tumor cells that line the vessels. Thus, NIR-PIT creates a potential space just outside blood vessels that increases leakage of nano-sized molecules into the extravascular space. Therefore, after the first NIR-PIT, circulating APC (or large molecules) can permeate deeper into the interstitium of the treated tumor making the second exposure to NIR-light more effective. Previous experiments suggested that the SUPR effect after NIR-PIT resulted in up to a 24-fold increase in the delivery of nano-sized molecules compared to baseline while also promoting more homogeneous redistribution of antibody-IR700 conjugates within the tumor after the initial NIR-PIT treatment [14]. In this study, Laser-light resulted in stronger SUPR effects than LED-light, which also increases the efficacy of NIR-PIT.

Orthotopic models used in other studies [15–17] are better for simulating cancer region in clinical patients than subcutaneous models used in this study. However, in this study, we focused on comparing two light emitting devices, a LED and a Laser system, for therapeutic effectiveness of NIR-PIT. From the physics point of view, a consistent size, shape and location of each tumor was necessary for fairly comparing these devices in an *in vivo* model that was difficult to achieve in technically complicated orthotopic models. Therefore, we used this simple subcutaneous tumor model in *in vivo* study.

In conclusion, Laser-light showed superior therapeutic tumor effects both in *in vitro* and *in vivo* mice models compared to LED at the same energy. In addition to direct enhancement of cytotoxicity, Laser-light promoted indirect enhancement of NIR-PIT by inducing a stronger SUPR effect in treated tissue. These findings could inform the choice of NIR-light source for human clinical trials.

## MATERIALS AND METHODS

### Reagents

Water soluble, silicon-phthalocyanine derivative, IRDye 700DX NHS ester and IRDye 800CW NHS ester were obtained from LI-COR Bioscience (Lincoln, NE, USA). Panitumumab, a fully humanized IgG_2_ mAb directed against EGFR, was purchased from Amgen (Thousand Oaks, CA, USA). All other chemicals were of reagent grade.

### Synthesis of IR700/IR800-conjugated panitumumab

Conjugation of dyes to panitumumab was performed according to previous reports [18, 19]. Panitumumab (1 mg, 6.8 nmol) was incubated with IR700 NHS ester (60.2 μg, 30.8 nmol) or IRDye 800CW NHS ester (35.9 μg, 30.8 nmol) in 0.1 mol/L Na_2_HPO_4_ (pH 8.6) at room temperature for 1 hr. The mixture was purified with a Sephadex G25 column (PD-10; GE Healthcare, Piscataway, NJ, USA). The protein concentration was determined with Coomassie Plus protein assay kit (Thermo Fisher Scientific Inc, Rockford, IL, USA) by measuring the absorption at 595 nm with spectroscopy (8453 Value System; Agilent Technologies, Santa Clara, CA, USA). The concentration of IR700 or IR800 was respectively measured by absorption at 689 nm or 774 nm with spectroscopy to confirm the number of fluorophore molecules conjugated to each mAb. The synthesis was controlled so that an average of three IR700 molecules and two IR800 molecules were bound to a single antibody. We performed SDS-PAGE as a quality control for each conjugate.

We abbreviate the IR700-panitumumab conjugate as pan-IR700, and the IR800-panitumumab conjugate as pan-IR800. Specific binding of pan-IR700 was confirmed with a blocking study *in vitro* (Supplementary Figure 1).

### Cell culture

EGFR-expressing A431 cells and MDA-MB-468-luc cells (stably luciferase-transfected) were used in these experiments. To evaluate specific cell killing by NIR-PIT, 3T3 cells stably expressing DsRed (3T3/DsRed) were used as a negative control [8]. Cells were grown in RPMI 1640 (Life Technologies, Gaithersburg, MD, USA) supplemented with 10% fetal bovine serum and 1% penicillin/streptomycin (Life Technologies) in tissue culture flasks in a humidified incubator at 37°C in an atmosphere of 95% air and 5% carbon dioxide.

### 3D spheroid culture

Spheroids were generated according to previous reports [7, 11, 20]. Spheroids were generated by the hanging drop method. Five thousand cells were suspended in 50 μL medium and were then dispensed into 96 well plates (3D Biomatrix Inc, Ann Arbor, MI, USA) following manufacture's instructions.

### Fluorescence microscopy

To detect the antigen specific localization of pan-IR700, fluorescence microscopy was performed (IX61 or IX81; Olympus America, Melville, NY, USA). Ten thousand cells were seeded on cover-glass-bottomed dishes and incubated for 24 hr. Pan-IR700 was then added to the culture medium at 10 μg/mL and incubated at 37°C. The cells were then washed with PBS; Cytox Blue (1:500) (Life Technologies) was used to detect dead cells [11]. Cytox Blue was added into the media 30 min before NIR-PIT. The cells were then exposed to NIR-light (2 J/cm^2^) and serial images were obtained. The filter was set to detect IR700 fluorescence with a 590–650 nm excitation filter, and a 665–740 nm band pass emission filter.

Analysis of the images was performed with ImageJ software (http://rsb.info.nih.gov/ij/).

### Flow cytometry

Fluorescence from cells after incubation with pan-IR700 was measured using a flow cytometer (FACS Calibur, BD BioSciences, San Jose, CA, USA) and CellQuest software (BD BioSciences). A431 and MDA-MB-468-luc cells (1 × 10^5^) were incubated with pan-IR700 for 6 hr at 37°C. To validate the specific binding of pan-IR700, excess panitumumab (50 μg) was used to block 0.5 μg of pan-IR700 (Supplementary Figure 1) [18].

### *In vitro* NIR-PIT

One hundred thousand cells were seeded into 24 well plates and incubated for 24 hr. Medium was replaced with fresh culture medium containing 10 μg/mL of pan-IR700 and incubated for 6 hr at 37°C. After washing with PBS, phenol red free culture medium was added. Then, cells were irradiated with either a red LED (L690-66-60; Marubeni America Co., Santa Clara, CA) or Laser (BWF5-690-8-600-0.37; B & W TEK INC., Newark, DE, USA). LED and Laser emit light at 670 to 710 nm and 685 to 695 nm wavelength, respectively. The power density was measured with an optical power meter (PM 100, Thorlabs, Newton, NJ, USA). To emit the same light dose (J/cm^2^) with either LED or Laser, the time of exposure was carefully adjusted.

### Cytotoxicity/phototoxicity assay

The cytotoxic effects of NIR-PIT with pan-IR700 were determined by the luciferase activity. The cytotoxic effects of NIR-PIT with pan-IR700 were determined by the luciferase activity. 150 μg/mL of D-luciferin-containing media (Gold Biotechnology, St Louis, MO, USA) was administered to PBS-washed cells 1 hr after PIT, and analyzed on a bioluminescence imaging (BLI) system (Photon Imager; Biospace Lab, Paris, France).

### LDH cytotoxicity assay

The cytotoxic effects of NIR-PIT on A431 spheroids were determined with the Cytotoxicity Detection Kit Plus (Roche Applied Science, Basel, Switzerland), which can detect cell membrane damage. Day 7 spheroids, pre-incubated with pan-IR700 for 6 hr, were washed with PBS, and transferred to 96 well plates (containing PBS), then irradiated with NIR-light using LED or Laser. One hr later after irradiation, the assay was performed. The analysis was done with a VICTOR-X3 plate reader (Perkin Elmer, Woodlands, TX, USA), and calculation of cytotoxicity was made according to manufacturer's instructions. All other procedures were performed following manufacturer's instructions.

### Animal and tumor models

All *in vivo* procedures were conducted in compliance with the Guide for the Care and Use of Laboratory Animal Resources (1996), US National Research Council, and approved by the local Animal Care and Use Committee. Six- to eight-week-old female homozygote athymic nude mice were purchased from Charles River (NCI-Frederick). During procedures, mice were anesthetized with isoflurane.

Two million A431 cells were injected subcutaneously in the right dorsum of the mice for evaluation of NIR-PIT effects on tumor volume and survival. In order to determine tumor volume, the greatest longitudinal diameter (length) and the greatest transverse diameter (width) were measured with an external caliper. Tumor volumes based on caliper measurements were calculated by the following formula; tumor volume = length × width^2^ × 0.5. Tumors reaching approximately 50 mm^3^ in volume were selected for the study. For fluorescence image and examination of SUPR effect two million A431 cells were injected subcutaneously in the right and left dorsum of the mice. Six million MDAMB468-luc cells were implanted into the right and left mammary fat pads for evaluation of cellular activity by BLI. D-luciferin (15 mg/mL, 200 μL) was injected intraperitoneally into mice 14 days after cell implantation, and analyzed with a Photon Imager for luciferase activity, and then mice were selected for further study based on tumor size and bioluminescence signals.

### *In vivo* NIR-PIT

A431 tumor-bearing mice (right dorsum tumor xenograft) were randomized into 5 groups of at least 10 animals per group for the following treatments: (1) no treatment (control); (2) only LED NIR-light exposure at 10 J/cm^2^ on day 1 and 20 J/cm^2^ on day 2; (3) only Laser NIR-light exposure at 10 J/cm^2^ on day 1 and 20 J/cm^2^ on day 2; (4) 100 μg of pan-IR700 i.v. every week, no NIR-light exposure; (5) 100 μg of pan-IR700 i.v. every week, LED NIR-light was administered at 10 J/cm^2^ on day 1 after injection and 20 J/cm^2^ on day 2 after injection; (6) 100 μg of pan-IR700 i.v. every week, Laser NIR-light was administered at 10 J/cm^2^ on day 1 after injection and 20 J/cm^2^ on day 2 after injection. These therapies were performed every week for up to 2 weeks. Mice were monitored daily, and tumor volumes were measured three times a week until the tumor diameter reached 2 cm, whereupon the mice were euthanized with carbon dioxide. For fluorescence imaging, A431 tumor bearing mice were injected with 100 μg of pan-IR700 and irradiated as follows: (1) Laser NIR-light was administered at 10 J/cm^2^ on day 1 after injection and 20 J/cm^2^ on day 2 after injection for tumor on the right side; (2) LED NIR-light was administered at 10 J/cm^2^ on day 1 after injection and 20 J/cm^2^ on day 2 after injection for tumor on the left side. Fluorescence images, as well as white light images, were obtained using a Pearl Imager with a 700 nm fluorescence channel. For analyzing fluorescence intensities, tumors of the same size were compared and regions of interest (ROI) were placed over the entire tumor. Average fluorescence intensity of each ROI was calculated. When comparing fluorescence target-to-background ratio (TBR), ROIs were placed in the adjacent non-tumor region. For the BLI study, mice were injected with 100 μg of pan-IR700 and irradiated as follows: (1) LED NIR-light was administered at 10 J/cm^2^ on day 1 after injection and 20 J/cm^2^ on day 2 after injection for tumor in the right side; (2) Laser NIR-light was administered at 10 J/cm^2^ on day 1 after injection and 20 J/cm^2^ on day 2 after injection for tumor in the left side. NIR-light exposure was performed 15 days after cell implantation. Mice images were acquired serially with a fluorescence imager (Pearl Imager) to detect IR700 fluorescence, and a Photon Imager for BLI. For analyzing BLI, an ROI of consistent size was placed over the entire tumor. When comparing fluorescence TBR, the average fluorescence intensity of each ROI was measured, and ROIs were placed in the adjacent non-tumor region (e.g. a symmetrical region to the left of the tumor). The calculation of TBR has been previously described [18].

### Evaluation of SUPR effect with pan-IR800

In order to investigate the vessel permeability and retention of macromolecular agents within the tumor after NIR-PIT, pan-IR800 was used as described previously [12]. Briefly, 1 hr after NIR-PIT (10 J/cm^2^) with the Laser directed at the right tumor and with LED directed at the left tumor in A431 xenografted mice and Laser directed at the left tumor and LED directed at the right tumor of the MDA-MB-468-luc orthotopic mouse model, 100 μg of pan-IR800 was intravenously injected, and imaging studies were performed at the indicated time points with a Pearl Imager using 700 nm and 800 nm channels. For analyzing fluorescence intensities, mean intensities of IR800 of each ROI were calculated.

### Statistical analysis

Data are expressed as mean ± s.e.m. based on a minimum of four experiments, unless otherwise indicated. Statistical analyses were carried out with GraphPad Prism (GraphPad Software, La Jolla, CA, USA). For multiple comparisons, a one-way analysis of variance (ANOVA) with post test (Kruskal-Wallis test with post-test) was used. Student's *t* test was also used to compare the two conjugates; *p* < 0.05 was considered to indicate a statistically significant difference.

## SUPPLEMENTARY MATERIALS FIGURE



## Abbreviations

PIT: photoimmunotherapy
IR700: IRDye-700DX
NIR: near-infrared
EGFR: epidermal growth factor receptor
pan: panitumumab
LED: light-emitting diode
Laser: light amplification by stimulated emission of radiation
SUPR: super-enhanced permeability and retention.
