# Supplementary material for: Comparative effectiveness of light emitting diodes (LEDs) and Lasers in near infrared photoimmunotherapy
Source: Oncotarget. 2016 Feb 13;7(12):14324–35. doi: 10.18632/oncotarget.7365 (PMC4924718; doi:10.18632/oncotarget.7365)
Supplement: Supplementary file 1 [file oncotarget-07-14324-s001.pdf]

Comparative effectiveness of light emitting diodes (LEDs) and lasers in near infrared photoimmunotherapy

Supplementary Materials

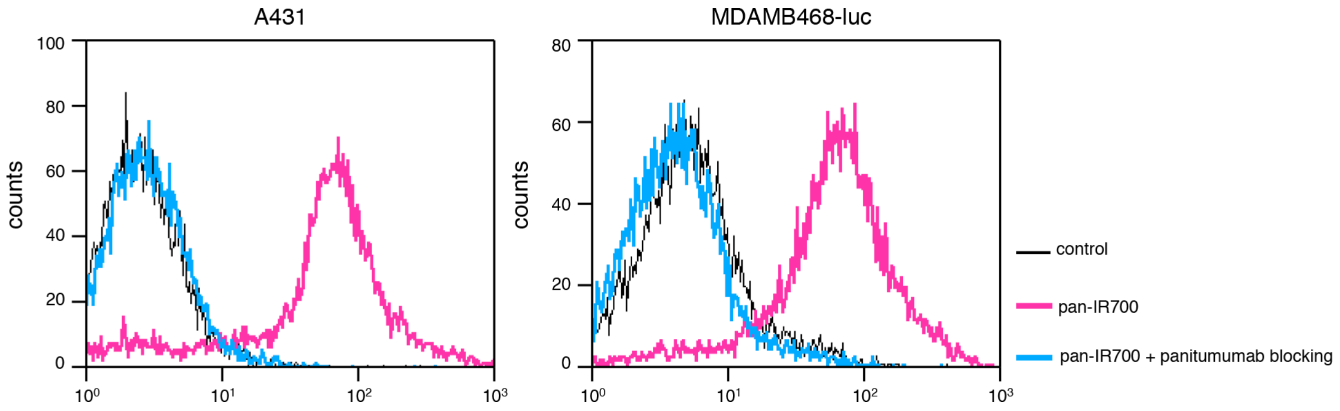

Supplementary Figure S1: Specific binding of pan-IR700 to EGFR in A431 cells and MDAMB468-luc cells. Specific binding function to EGFR on either cell-lines was demonstrated by flow cytometry.
